# Are Biodegradable Osteosyntheses Still an Option for Midface Trauma? Longitudinal Evaluation of Three Different PLA-Based Materials

**DOI:** 10.1155/2015/621481

**Published:** 2015-09-27

**Authors:** Andreas Kolk, Robert Köhnke, Christoph H. Saely, Oliver Ploder

**Affiliations:** ^1^Department of Oral- and Craniomaxillofacial Surgery, Klinikum Rechts der Isar der Technischen Universität München, Ismaninger Strasse 22, 81675 Munich, Germany; ^2^Department for Oral- and Maxillofacial Surgery, University Medical Center Hamburg-Eppendorf, Martinistrasse 52, 20246 Hamburg, Germany; ^3^Department of Oral- and Maxillofacial Surgery, Academic Teaching Hospital Feldkirch, Carinagasse 47, 6800 Feldkirch, Austria; ^4^Department of Medicine and Cardiology, VIVIT Research, Academic Teaching Hospital, Carinagasse 47, 6800 Feldkirch, Austria; ^5^Department of Craniomaxillofacial and Oral Surgery, Medical University Vienna, Vienna General Hospital, Waehringer Guertel 18-20, 1090 Vienna, Austria

## Abstract

The aim was to evaluate three different biodegradable polylactic acid- (PLA-) based osteosynthesis materials (OM). These OM (BioSorb, LactoSorb, and Delta) were used in 64 patients of whom 55 (85.9%) had fractures of the zygoma, five (7.8%) in the LeFort II level, two of the frontal bone (3.1%), and two of the maxillary sinus wall (3.1%). In addition to routine follow-up (FU) at 3, 6, and 12 months (m) (T1, T2, and T3) all patients were finally evaluated at a mean FU after 14.1 m for minor (e.g., nerve disturbances, swelling, and pain) and major (e.g., infections and occlusal disturbances) complications. Out of all 64 patients 38 presented with complications; of these 28 were minor (43.8%) and 10 major (15.6%) resulting in an overall rate of 59.4%. Differences in minor complications regarding sensibility disturbance at T1 and T3 were statistically significant (*P* = 0.04). Differences between the OM were not statistically significant. Apart from sufficient mechanical stability for clinical use of all tested OM complications mostly involved pain and swelling probably mainly related to the initial bulk reaction attributable to the drop of pH value during the degradation process. This paper includes a review of the current aspects of biodegradable OM.

## 1. Introduction

In maxillofacial trauma, osteosynthesis materials (OM) manufactured from titanium have been routinely used for many years [1, 2]. Such bone plates are biocompatible and provide adequate stability. Several potential problems with these systems can occur including palpability, temperature sensitivity, infection, interference with radiographic imaging [3, 4] and radiation therapy, and the necessity of removal especially in the young growing face after 3–6 months (m) [5]. Additionally, scar tissue covering these plates and locoregional lymph nodes can contain titanium particles [6]. In a recent publication, titanium plates have even been seen as a risk factor for the development of the bisphosphonate-related osteonecrosis of the jaw [7]. In order to avoid these problems, biodegradable synthetic semicrystalline polymers that are mainly polylactic acid- (PLA-) based have been developed for use as OM in maxillofacial trauma [8, 9]. Since their first descriptions in the 1970s and mainly within the last 20 years, biodegradable OM have been investigated and shown to achieve adequate strength, rigidity, and biocompatibility [10–14]. Initially, synthetic polymers of lactic (PLA) and glycolic (PGA) acid were considered to be biocompatible and rigid [15], with PLA also serving as a device for controlled drug delivery in addition to its use as an OM [16, 17]. Because of various problems with OM based only on PGA, and because of the early loss of stability attributable to fast degradation [18], further developments have concentrated more on the high-molecular-weight biodegradable polymer PLA, a combination of two different stereoisomeric forms such as poly-L-lactide (PLLA) and poly-D-lactide (PDLA), or a combination of PLA and PGA. The last-mentioned form is characterized by an inferior degradation rate attributable to lower crystallinity and minor resistance against hydrolysis. To combine properties, copolymers of PLA are joined in different ratios of PLLA and PDLA.

Thus, many different materials with diverse compositions of PLA for various applications in the field of oral and maxillofacial surgery are available on the market and are used in the treatment of fractures of the frontal bone and midface [19, 20]. Although many of these materials are widespread, no evidence has been presented for their indication and localization, or whether they can serve as an alternative to titanium-based OM. Most problems with biodegradable OM are related to the duration of the degradation process with a consecutive change of the local tissue environment caused by foreign body reaction and tissue shrinking mainly within weeks, but also up to many months after implantation.

The vast majority of clinical studies have compared the outcome of the use of biodegradable materials with titanium in the treatment of midface fractures [21–24]. The results of the latter studies have shown no differences between biodegradable and titanium fixation regarding short-term outcome. Little long-term data are available concerning comparisons of various biodegradable materials in clinical applications [21, 25]. Therefore, the aim of this study has been to evaluate the use of three different biodegradable OM in the treatment of midface trauma and to analyze their long-term clinical outcome.

## 2. Patients and Methods

Over a period of 45 months, 64 patients (50 men and 14 women, mean age (±SD) 30.2 ± 15.4 years, ranging from 6 to 80 years) with fractures of the midfacial skeleton were enrolled in the study and, after randomization, were treated at the University Hospital of Cranio-Maxillofacial and Oral Surgery of the Medical University Vienna with three different biodegradable OM as an alternative to the classic titanium OM: BioSorb (copolymer with PLLA/PDLA (ratio 70 : 30), Bionx Implants Linvatec Corp., Largo, FL, USA) (BS), LactoSorb (amorphic copolymer with PLLA/PGA (ratio 82 : 18), Walter Lorenz Surgical, Inc., Jacksonville, FL, USA) (LS), and Delta (terpolymer with PLLA/PDLA/PGA (ratio 85 : 5 : 10), Stryker Leibinger Micro Corp., Freiburg, Germany) (DS).

**Figure 1:**
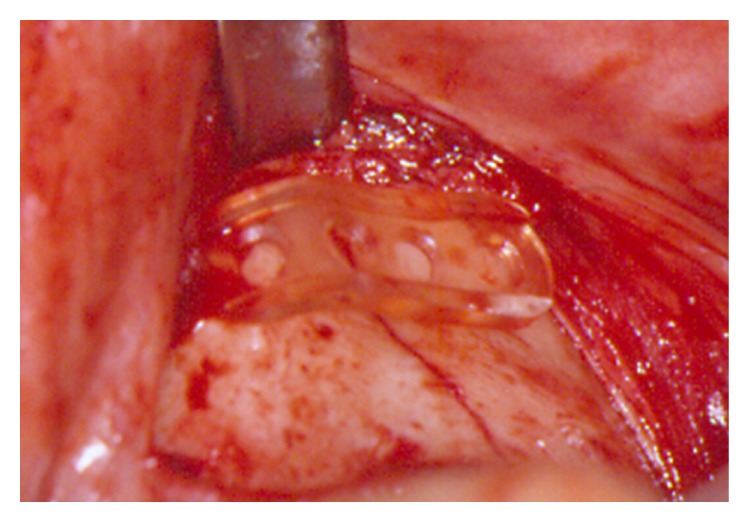
Intraoperative view. Adaption of biodegradable OM in the zygoma region.

In contrast to the two other OM (LS/DS), BS plates on the basis of a self-reinforced poly (L-/DL-) (70 : 30) lactic acid copolymer can be adapted to the bony contour (Figure 1) at room temperature without any heating. To obtain a preferably homogeneous collective, patients were selectively filtered for this study. Subjects with previous surgery or systemic diseases, such as diabetes or osteoporosis, were excluded. After choosing biodegradable OM as an alternative to titanium, all patients gave their written informed consent for the surgical procedure and research purposes at the time of initial presentation. The local hospital ethic committee approved the study. Three, six, and twelve m after trauma (time points T1, T2, and T3), patients took part in routine follow-up (FU) with clinical and radiological examination (conventional X-rays or CT scans) by one investigator using a standardized FU protocol. Preoperative data were taken from the charts of each patient. All findings (temporary and permanent symptoms) that occurred during any part of the observation period were recorded and categorized as minor (e.g., swelling, pain, or nerve disturbance) or major (infection, occlusal disturbance, need of revision surgery, or hypertrophic scar) complications (Table 1). Parameters such as swelling and redness were recorded with yes/no answers. Pain was documented on a visual analog scale (VAS > 0) from 0 to 10. All the last-mentioned parameters were classified as minor complications if they were still observed at investigation date T1–T3. Potential dysfunctions (an-, hyp-, and paraesthesia) of the trigeminal nerve branches were tested on both sides at the forehead, cheek, nose, and lip area by using the touch-detection-threshold at T1–T3. Further postoperative findings such as infections were recorded. Maximum mouth opening and occlusal conditions (centric occlusion, lateral excursions, and occlusal disturbance) were analyzed in cases of occlusal involvement of the central midface (Table 1). At the end of the study after a mean (±SD) of 14.1 ± 0.8 m a final clinical examination took place in addition to the previous FU at T3.

Descriptive statistics for quantitative variables are given as means ± standard deviation and, where appropriate, as medians and ranges. All data were analyzed with the “Statistical Package for the Social Sciences” (SPSS for windows, release 14.0.0 2011; SPSS Inc.). All *P* values given are unadjusted, two-sided, and subject to a significance level of *P* < 0.05.

## 3. Results

In 64 patients, the fractures were treated with three different biodegradable OM (BS, LS, and DS); 55 patients (85.9%) had zygoma fractures, five patients (7.8%) had fractures in the LeFort II level, two of the frontal bone (3.1%), and two of the maxillary sinus wall (3.1%) (Table 2). Thirty-six patients (56.2%) were treated with BS, 12 patients (18.8%) were treated with LS, and in 16 cases (25%) DS was used. Fracture localization, the number of cases in each area, and the OM used are shown in detail in Table 2. The mean time interval (±SD) between trauma and operation was 3.9 ± 4.8 days. Patients were divided into two groups regarding the time duration to fracture reduction: one group was operated up to day 4 after injury and the other between 5 and 9 days after trauma. The difference between the two groups was not statistically significant. The mean final clinical FU (±SD) was after 14.1 ± 0.8 m (ranging from 12.4 to 22 m). All fractures showed stable healing without any signs of redislocation until T1. None of the patients required any revision surgery or other additional procedures.

Out of the total collective (*N* = 64) 38 patients presented with problems, of these 28 (43.8%) had minor and 10 (15.6%) major complications resulting in an overall complication rate of 59.4%. Whereas differences regarding sensory disturbance between T1 and T3 were statistically significant (*P* = 0.04), the variances in the outcome between the materials were statistically not significant (Tables 3 and 4). Twelve months postoperatively (T3), 37 patients (57.8%) experienced pain in the plate region (VAS > 0): out of the latter subgroup, 28 patients belonged to group BS (77.8%), two to group LS (16.7%), and seven to group DS (43.8%). The difference between the materials was not statistically significant. At the final clinical investigation date (mean 14.1 m postoperatively), pain (VAS > 0, mean ± SD, 3.05 ± 1.31) was still present and was considered as quality-of-life-limiting complaints in 19 patients of group BS, one of group LS, and 6 of subgroup DS. Differences between the materials were statistically marginally significant (*P* = 0.05).

In 18 patients treated with BS (50.0%), in three patients of group LS (25.0%), and in five patients with DS (31.3%), swelling was still evident at the final clinical evaluation time point (after mean 14.1 m). The differences between the materials were statistically not significant. The presence of nerve disturbance and its different degrees of severity or quality, the fracture localization, and the applied OM subgroup are displayed in Tables 3 and 4.

## 4. Discussion

**Figure 2:**
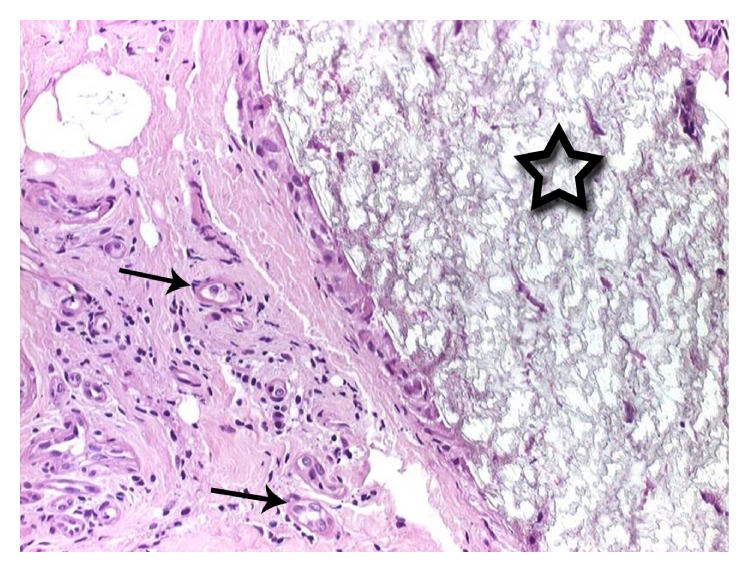
Histologic evaluation of a biodegradable material (asterix). Surrounding foreign body reaction with giant-cell-formation (arrows).

The use of titanium plates and screws for the treatment of facial fractures is well documented and accepted as the treatment of choice [1, 2]. In order to avoid implant-related problems with these materials, for example, palpable and prominent plates or thermal sensitivity followed by a second operation for removal, biodegradable OM were invented for the treatment of facial fractures more than 40 years ago [10, 12, 26–30]. Additionally, these materials are used for craniofacial and reconstructive facial surgery [23, 30, 31]. Nevertheless, only a few studies have compared outcome with regard to the localization and different material compositions of the OM [22, 24, 25, 32]. The complication rate in these investigations varies between 0.0% and 22.8% [22, 24, 31, 32]. Eppley et al. have reported their experience and success with L-/DL-lactide (70/30) for the fixation of maxillofacial trauma, including fractures of the central midface, zygoma, and orbital rim and floor [23]; they observed no implant-related complications (e.g., infection, erythema, fracture instability, or relapse) up to one year after fixation. Enislidis et al. have recorded 22.8% of minor complications by using BioSorb (BS) for the fixation of zygoma fractures [24]. In contrast to the current literature and an analysis of the latter, we have obtained a much higher complication rate of 59.4%, independent of the material. This can be explained by the long-term FU and the extensive listing of various clinical symptoms, including swelling, being categorized as major or minor complications. Furthermore, the evaluation of swelling and pain have been categorized with yes or no answers and assigned to minor complications, for example, each patient presenting with swelling (yes or no answers) or pain (VAS > 0 equals pain). The majority of minor complications are related to pain and swelling. At T3 even 37/64 patients (57.8%) and even at the final FU 26 of 64 patients (40.6%) still exhibited pain and swelling in the plate region. The high number of these symptoms can probably be explained by implant-related foreign body reaction (Figure 2) caused by the material-associated degradation process and the thickness of the plates (Figure 1) in general with local tissue trauma. Bioresorbable polymers are mainly high-molecular-weight aliphatic polyesters with repeating units of *α*-hydroxy acid (HO- CHR-COOH) derivatives manufactured by ring-opening polymerization [33]. The absorption of these polymers begins with depolymerization through the acid hydrolysis of their ester bonds. The local pH value drops followed by a change in osmotic pressure. Toxic responses result [34] in concomitant damage of macrophages and fibroblasts [35], and osteoblasts are affected [36]. The resulting chronic inflammatory response by the body leads to acid hydrolytic degradation [37–39]. The material is probably metabolized by macrophages via the citric acid cycle and converted into CO_2_ and H_2_O via bulk hydrolysis; it is also metabolized by the liver (a two-phase degradation process) [40, 41].

The resorption time of the metabolized components ranges between 6 and 18 months and can even occur up to 60 months [42, 43]. Generally, degradation characteristics depend on many factors of the material itself and the local tissue environment including the copolymer ratio, micro- and molecular structure, processing conditions, implant shape and thickness, and implantation site including vascularization. Initial biodegradable systems involved high-molecular-weight polylactic acid polymers, but unfortunate foreign body reactions occurred and were attributed to their long resorption period [44, 45]. BS, for example, is a copolymer formed by combining L-lactide and DL-lactide (70 : 30) to provide optimal strength and acceptable spatiotemporal degradation characteristics. It retains approximately 68% of its initial bending strength after 8 weeks, approximately 30% after 6 months, and has a total resorption time of 24 months [32]. The copolymer compositions and thus the degradation behavior of LS + DS differ slightly from that of BS. Despite their possible differences in stability during the fracture healing process and in the local tissue environment attributable to the different rates in the pH value decrease, only minor differences concerning the clinical outcome were apparent between BS, LS, and DS. BS exhibited slightly more long-term swelling and pain, as their overall degradation time span was longer than those of LS and DS. In the literature, some serious nonspecific foreign body reactions have been reported as being caused by high-molecular PLLA implants [44, 46, 47]. The PLLA remnants are surrounded by a fibrotic capsule and have been detected intracellularly. Even after 5.7 years, unabsorbed PLLA particles are present in the specimens [44, 46, 47].

Another important aspect is the ability of the implantation site to dissolve and remove metabolized materials. Good vascularization leads to faster removal and prevents the accumulation of degradation products causing acidity of the tissue [48, 49].

After the first week of implantation, a rapid decline in strength of PLLA occurs, which might lead to premature failure [50]. A 50% loss of strength by two weeks after implantation and a total loss of strength and consistency after 6 weeks limit the reliable clinical use of this material [50]. In an experimental study, four biodegradable materials (LactoSorb, Inion CPS 1.5 baby, Delta, and RFS) were evaluated, and their stability was tested* in vitro* via microrigidity [51]. LactoSorb and Inion CPS 1.5 baby were the weakest implants after three months. After a year, Delta and RFS were still rigid [51]. Biodegradable materials are composed of various combinations of poly(*α*-hydroxypolyesters), such as polylactic acid (PLA) and polyglycolic acid (PGA), and therefore show diverse intensities of inflammatory reaction because of their variable degradation rates [30, 52, 53]. Intraoperative warming of most OM (not BS) is needed in order to adapt the shape of the biodegradable material to the anatomical implantation site. Bergsma et al. speculate that this manipulation accelerates the degradation progress [49]. Furthermore, crystallinity leads to a slower absorption rate and is known to cause greater tissue reaction than with more easily absorbed components [48]. In future, polymethylmethacrylate bone cement bonding and degradable magnesium alloy implants [54, 55] might alternatively be used for the treatment of facial trauma, especially in the non-load-bearing region of the frontal and calvaria bone [56].

In our study, we have found a large number of complications such as pain, swelling, infections, and nerve disturbances. Most of the documented problems are related to the implantation site and not to the materials. However, we assume that the degradation process of the implants is another important reason for the high number of minor complications. Because of the inevitable drop of the local pH-value, short- and long-term effects occur as the osmotic pressure is increased, so that the implant cavity is expanded or sterile fluid accumulates.

The shrinking of the periosteum, pain, and sensory disturbance, sometimes over many months, are probably the most important negative consequences for the patient. Buffering systems such as the incorporation of basic salts [57] and other modifications might reduce these unpleasant side effects and might therefore help to broaden the range of applications of these OM in the field of facial trauma and reconstruction and to increase patient acceptance. Many factors influence the physicochemical behavior and, consecutively, the degradation process of PLA [17]. Whereas low stress, high crystallinity, and orientation can reduce the degradation rate [58]; high temperature [59] and acidity [60] tend to induce the opposite.

However, further investigations with modified copolymer compositions, buffering systems, and biodegradable OM with more tissue-compatible physicochemical characteristics thus need to be carried out and analyzed.

In conclusion, the current study and the literature provide evidence that the use of biodegradable OM in the treatment of fractures of the midface and particularly in load-bearing applications such as the mandible is still not an alternative to the classic titanium OM and should therefore be reserved for specific indications. Moreover, long-term effects generated by the degradation process and its products have to be critically observed. Developments such as degradable magnesium alloy implants [54] might show stability comparable with titanium, might be an alternative to the latter, and might replace classic biodegradable OM.

## Figures and Tables

**Table 1:** Categories of potential complications.

Minor complications	Major complications
Swelling^*∗*^	Infection

Redness^*∗*^	Malocclusion

Pain^*∗*^	Revision surgery

Sensitivity disturbance	Ectropion
	Hypertrophic scar

^*∗*^Recorded if still observed at the final clinical investigation date (mean final FU after 14.1 m).

**Table 2:** Distribution of fracture localization and applied OM (*N* = 64).

Localization	Number	Material		
		BS	LS	DS
Frontal bone	2	2 (1.5)		
Midface				
Le Fort II	5	5 (2.0)		
Zygoma	55	27 (1.5)	12 (1.5)	16 (1.7)
Max. sinus wall	2	2 (1.5)		

Total	64	36	12	16

OM with plate thickness behind the number (mm); BS: Biosorb; LS: LactoSorb; DS: Delta.

**Table 3:** Fracture localization and distribution and quality of nerve disturbance (*N* = 64).

FU	Localization	Norm (%)	Hyp (%)	Par (%)	An (%)	MD (%)	*P*
T1	Upper jaw	11 (17.2)	43 (67.2)	7 (10.9)	3 (4.7)	0	
T2	Upper jaw	35 (54.7)	26 (40.6)	2 (3.1)	1 (1.6)	0	
T3	Upper jaw	50 (78.1)	8 (12.5)	4 (6.3)	1 (1.6)	1 (1.6)	0.045^*∗*^

FU: Follow-up time point in months (m), (T1 = 3 m, T2 = 6 m, T3 = 12 m); Norm: normal; Hyp: hypaesthesia; Par: paraesthsia; An: anaesthesia; MD: missing data. Apart from T1 versus T3, no statistical significance was seen between the FU time points, level of significance ^*∗*^(*P* < 0.05).

**Table 4:** Nerve disturbance depending on FU and OM (*N* = 64).

FU	OM	Norm	Hyp	Par	An	MD	*P*
T1	BS	5 (13.9%)	25 (69.4%)	3 (8.3%)	3 (8.3%)	0	—
	LS	2 (16.7%)	10 (83.3%)	0	0	0	—
	DS	4 (25.0%)	8 (50.0%)	4 (25.0%)	0	0	—

T2	BS	14 (38.9%)	14 (38.9%)	2 (5.6%)	1 (2.8%)	5 (13.9%)	—
	LS	9 (75.0%)	3 (25.0%)	0	0	0	—
	DS	11 (68.8%)	3 (18.8%)	2 (12.5%)	0	0	—

T3	BS	23 (63.9%)	5 (13.9%)	2 (5.6%)	1 (2.8%)	5 (13.9%)	—
	LS	10 (83.3%)	2 (16.7%)	0	0	0	—
	DS	12 (75.0%)	2 (12.5%)	2 (12.5%)	0	0	—

FU: FU time point (T1 = 3 m, T2 = 6 m, and T3 = 12 m); Norm: normal; Hyp: hypaesthesia; Par: paraesthesia; An: anaesthesia; MD: Missing Data. *P*: the difference between the materials was not statistically significant.

## Abbreviations

FU: Follow-up
m: Months
OM: Osteosynthesis material
PLA: Polylactic acid
PGA: Polyglycolic acid
PDLA: Poly-D-Lactic Acid
PLLA: Poly-L-Lactic Acid
T: Time point.
